# ‘Gardos Channelopathy’: a variant of hereditary Stomatocytosis with complex molecular regulation

**DOI:** 10.1038/s41598-017-01591-w

**Published:** 2017-05-11

**Authors:** Elisa Fermo, Anna Bogdanova, Polina Petkova-Kirova, Anna Zaninoni, Anna Paola Marcello, Asya Makhro, Pascal Hänggi, Laura Hertz, Jens Danielczok, Cristina Vercellati, Nadia Mirra, Alberto Zanella, Agostino Cortelezzi, Wilma Barcellini, Lars Kaestner, Paola Bianchi

**Affiliations:** 1 UOC Oncoematologia, UOS. Fisiopatologia delle Anemie Fondazione IRCCS Ca’ Granda Ospedale Maggiore Policlinico, Milano, Italy; 2 Vetsuisse Faculty and the Zurich Center for Integrative Human Physiology (ZIHP), Institute of Veterinary Physiology, University of Zurich, Zurich, Switzerland; 3 Research Center for Molecular Imaging and Screening, Medical School, Institute for Molecular Cell Biology, Saarland University, Homburg/Saar, Germany; 4 UOC Pronto soccorso, Pediatria ambulatoriale e DH/MAC. Fondazione IRCCS Ca’ Granda Ospedale Maggiore Policlinico, Milano, Italy; 5 Universita’ degli Studi di Milano, Milano, Italy; 6 Experimental Physics, Saarland University, Saarbruecken, Germany; 7 Theoretical Medicine and Biosciences, Saarland University, Homburg/Saar, Germany

## Abstract

The Gardos channel is a Ca^2+^ sensitive, K^+^ selective channel present in several tissues including RBCs, where it is involved in cell volume regulation. Recently, mutations at two different aminoacid residues in *KCNN4* have been reported in patients with hereditary xerocytosis. We identified by whole exome sequencing a new family with two members affected by chronic hemolytic anemia carrying mutation R352H in the *KCNN4* gene. No additional mutations in genes encoding for RBCs cytoskeletal, membrane or channel proteins were detected. We performed functional studies on patients’ RBCs to evaluate the effects of R352H mutation on the cellular properties and eventually on the clinical phenotype. Gardos channel hyperactivation was demonstrated in circulating erythrocytes and erythroblasts differentiated *ex*-*vivo* from peripheral CD34+ cells. Pathological alterations in the function of multiple ion transport systems were observed, suggesting the presence of compensatory effects ultimately preventing cellular dehydration in patient’s RBCs; moreover, flow cytometry and confocal fluorescence live-cell imaging showed Ca^2+^ overload in the RBCs of both patients and hypersensitivity of Ca^2+^ uptake by RBCs to swelling. Altogether these findings suggest that the ‘Gardos channelopathy’ is a complex pathology, to some extent different from the common hereditary xerocytosis.

## Introduction

The Gardos channel is a Ca^2+^ sensitive, intermediate conductance, K^+^ selective channel present in several cell types including red blood cells (RBCs)^1^, where it is involved in cell volume regulation. Activation of the channel in response to elevation of cytosolic Ca^2+^ in human erythrocytes causes transient cell shrinkage due to the efflux of K^+^ and concomitantly of Cl^−^, a phenomenon referred to as Gardos effect^2^. Patch-clamp experiments have shown that local membrane deformations may act as a stimulating event leading to Gardos channel activation in RBCs, providing evidence for the role of this mechanosensory mechanism in shaping and volume modifications of erythrocytes^3^.

In the last years Gardos channel has been identified as an interesting therapeutic target in human diseases^4, 5^; in particular, its inhibition in sickle cell disease patients has shown to reduce RBC dehydration and hemolysis, and to increase hemoglobin levels despite the lack of any reduction in the frequency of pain episodes^6–8^.

Gardos channel (KCa3.1) is a tetramer of 4 identical subunits, encoded by the *KCNN4* gene^9^. Recurrent mutations at two different aminoacid residues in *KCNN4* (R352H, V282M/E) have been reported in patients from 6 independent families with dehydrated hereditary stomatocytosis (DHSt)^10, 11, 12^. In a recent paper aimed at studying the effect of the Gardos channel inhibitor Senicapoc, it was observed that the three mutants result in a higher channel activity, although they do not share a common mechanism in altering channel characteristics, i.e. Ca^2+^ sensitivity^13^. However, the link of the Gardos channel dysfunction to increased hemolysis has so far not been elucidated.

To get a mechanistic link between the Gardos channel mutation, the cellular properties and eventually the clinical phenotype, we studied two novel patients carrying KCNN4 R352H mutation performing the following investigations: (a) single cell patch-clamp recordings on both RBCs and RBCs precursors, (b) measure of the activity of single ion transporters using ^86^Rb^+^ as a tracer for K^+^ flux experiments, (c) evaluation of intracellular ions contents and RBC glycolysis (d) Ca^2+^ handling by fluorescence live imaging and flow cytometry on RBCs. We found pathological alterations in the functions of multiple ion transport systems, and metabolic glycolytic impairment.

## Results

### Hematological data

The proband (II.4), a 40 years old man of Northern Italian origin, was referred to our Centre for the first time at the age of 3 months for evaluation of hemolytic anemia and hepatosplenomegaly; the unrelated parents and three siblings were hematologically normal. Hb levels ranged 7–9 g/dL, reticulocytes 250–350 × 10^9^/L, osmotic fragility was decreased or normal, no defects of RBC enzymes were detected. Bone marrow (BM) analysis revealed erythroid hyperplasia, and measurement of RBC survival showed reduced lifespan with intra-splenic hemolysis. The patient was occasionally transfused during spontaneous hemolytic crises; at the age of 11 he underwent splenectomy and cholecystectomy at 13. After splenectomy Hb levels were maintained around 10 g/dL, no further transfusions were required, thromboembolic events never occurred. Fibroscan, Magnetic Iron Detector (MID) and liver iron concentration showed moderate iron overload, consequently iron chelation was started.

Proband’s first daughter (III.1), born at term after an uneventful pregnancy, presented severe anemia requiring RBC transfusion at birth (Hb 6.1 g/dL) and at 3 months. Afterwards, until the age of 2 years, Hb levels stabilized to about 10 g/dL with no need of further transfusions. Her mother was hematologically normal.

Clinical and hematologic data at the time of the study are reported in Table 1. Both patients displayed moderate hemolytic anemia, reticulocytosis and abnormal RBC morphology with marked anisopoikilocytosis and stomatocytosis. BM examination in II.4 revealed erythroid hyperplasia with some dyserythropoietic changes, in particular binucleated erythroblasts (Supplementary Figure 1).

**Table 1:** Clinical and hematologic data of the patients at the time of the study.

	II.4	III.1	Ref values
Gender	M	F	
Age (yrs)	40	1	
Transfusions	occasional until splenectomy	2 (at birth and 3 month)	
Splenectomy (age)	yes (12)	no	
Thrombotic events	no	no	
Hb (g/dL)	10.5	9.9	13.4–17.5/10.5–14.5*
MCV (fL)	108	81.5	80–94/70–91*
MCH (pg)	35.8	29.1	25–35/24.5–31.5*
MCHC (g/dL)	33	35.7	31–37
Reticulocytes (×10^9^/L)	384	273	20–100
Erythroblasts (%)	8.6	n.a.	
RBCs morphology	13% stomatocytes	8% stomatocytes	
	7% echinocytes	3% elliptocytes	
	6% schistocytes	3% spherocytes	
	5% elliptocytes	2% schistocytes	
	4% spherocytes		
Unconj. bilirubin (mg/dL)	1.56	1.1	<1
Serum ferritin (ng/mL)	1108	97	30–400/15–150*
Plasma Na^+^ (mE/L)	139	137	135–145
Plasma K^+^ (mE/L)	4.1	4	3.3–5.1
RBC K^+^ (μmoles/gDW)	287.97	306.42	283.55
AGLT	55	>900	>900
Pink test	36	53	11–33
NaCl osmotic fragility	normal	increased	
EMA binding test	normal	normal	
Sp/B3 ratio	0.84	0.97	0.95–1.17
Abnormal hemoglobins	absent	absent	

n.a. = not available.

Sp = spectrin; B3 = band 3.

*Age dependent reference values.

### RBCs properties

**Figure 1:**
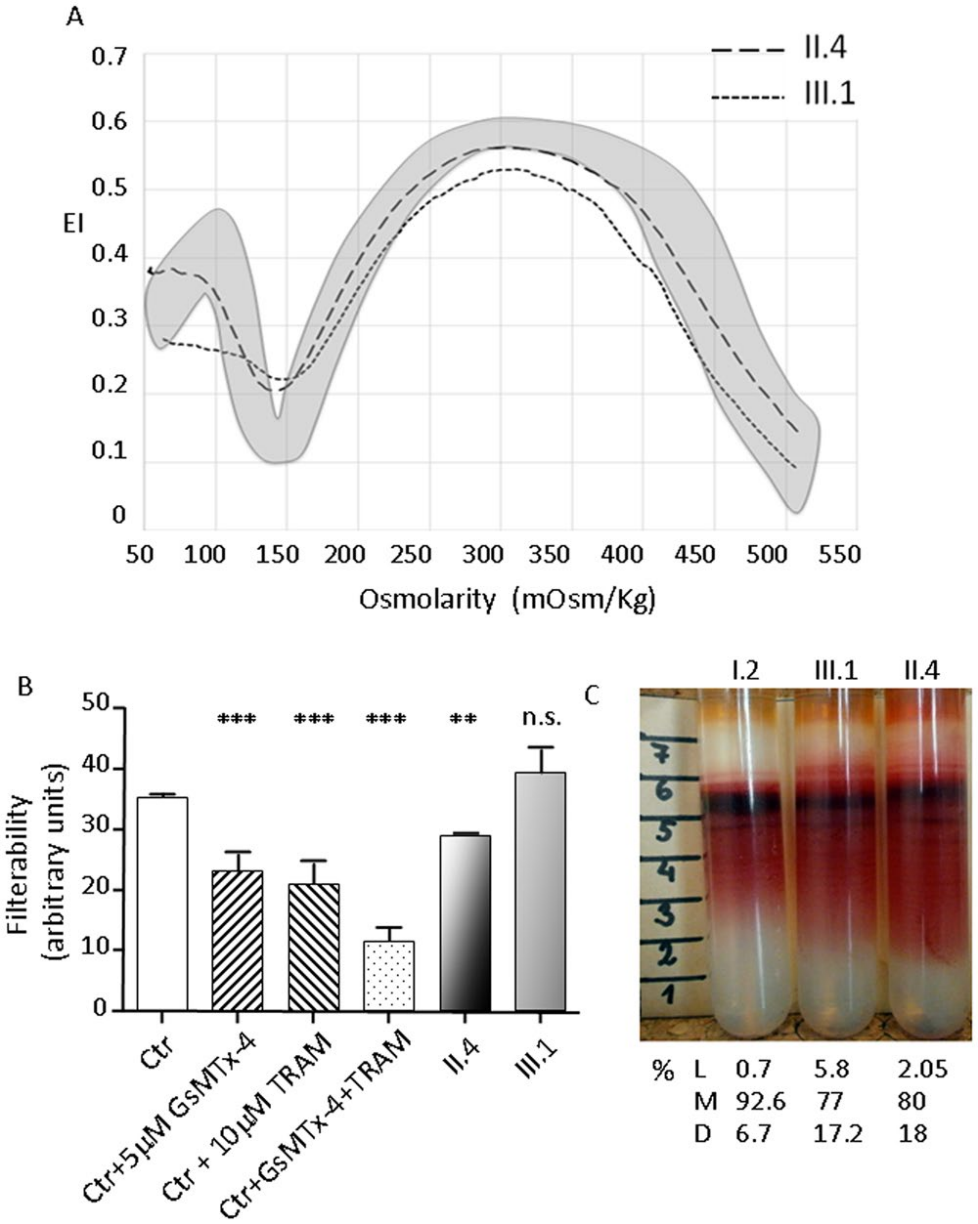
(**A**) LoRRca Osmoscan profile (grey area refers to 150 normal controls); (**B**) Filterabilty results; healthy cell untreated (Ctr, white bar), pre- incubated with GsMTx-4 (upward hatched bar), TRAM-34 (downward hatched bar) GsMTx-4 + TRAM-34 (dotted bar); patient II.4 (black shaded bar); patient III.1 (grey bar). All measurement were performed at least twice and significance was testes using an unpaired t-test. ** and ***denote p < 0.01 and 0.001 respectively; (**C**) Separation of RBC on Percoll density gradient and % of cells in low density (L), medium (M) and high (D) density fractions.

The osmotic gradient ektacytometry did not display the leftward shift of the curve typical of DHSt in both patients (O_min_ 142 and 149 respectively for II.4 and III.1, n.v. 136–152; O_Hyper_ 457 and 437, n.v. 446–474) (Fig. 1A), in spite of the presence of stomatocytes in the peripheral blood smear. No differences were observed by performing Osmoscan curve in different conditions (fresh blood, 24 h incubation at 4 °C) and with different anticoagulants (heparin, EDTA).

The RBCs filterability was decreased in patient II.4 (splenectomized) and normal in III.1 (not splenectomized, infant). To verify that cells needed to deform in this procedure, we compared untreated healthy cell with cells where mechano-sensitive channels were inhibited by preincubation with 5 µM GsMTx-4. To assess the contribution of the Gardos channel in filterability we pretreated cells with 10 µM of the Gardos-channel inhibitor TRAM-34. Both treatments resulted in a significant decrease in filterability compared to untreated controls. Pre incubation with 5 µM GsMTx-4 and TRAM-34 together caused further decrease in filterability (Fig. 1B).

Percoll density gradient separation showed an increased high-density cell fraction in both patients; medium-density fraction was shifted somewhat upwards in patient II.4 indicating an increase in MCV and a decrease in the average RBC density (Fig. 1C). Plasma of patients’ blood samples contained free hemoglobin. Hemolysis was most pronounced in II.4 sample showing disturbed filterability.

Taken together, the above reported results, in particular Osmoscan and filterability data, indicate that despite presence of stomatocytes, patients’ RBC didn’t show the expected dehydration. The slight differences in RBCs properties observed between III.1 and II.4 could be attributed to infant/adult age of the two patients and presence/absence of the spleen.

### Molecular analysis

**Figure 2:**
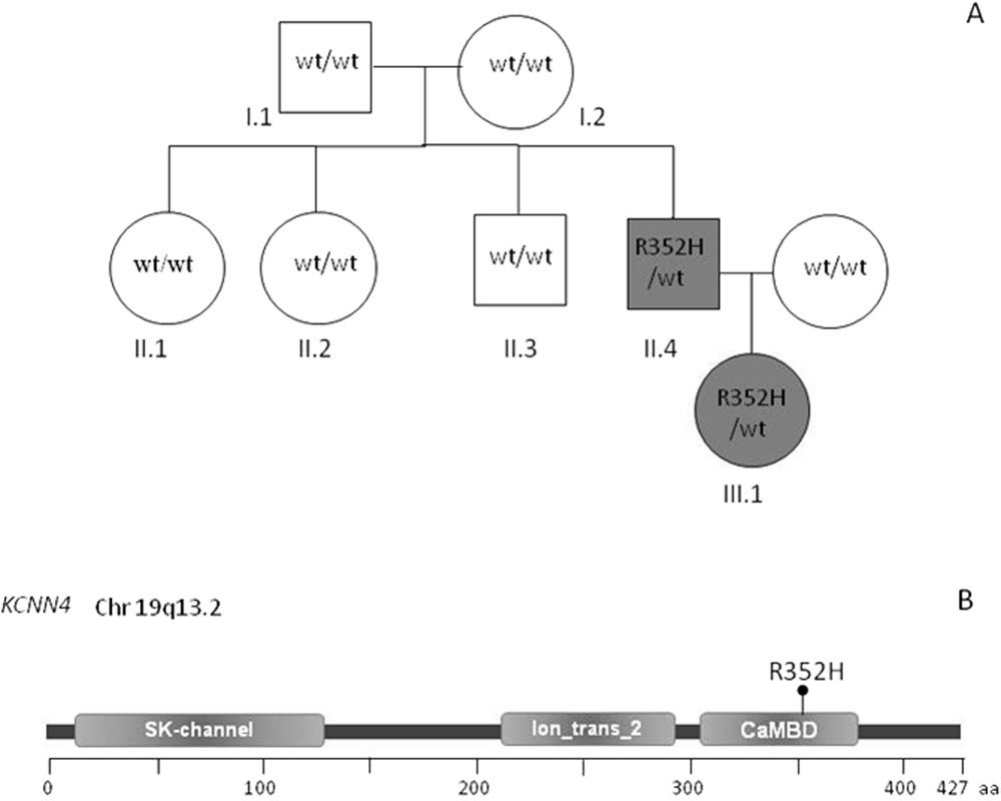
(**A**) Inheritance pattern of the family. (**B**) Schematic representation of KCNN4 protein domains, and position of R352H mutation.

All the family members underwent whole exome sequencing. Only one candidate gene (*KCNN4*) was identified by non-synonymous heterozygous mutations with expression in hematopoietic tissues. A *de novo* heterozygous missense mutation (c.1055G > A, p.R352H) was detected in II.4 and dominantly transmitted to the daughter III.1 (Fig. 2A). The mutation falling in exon 7 is involved in the calmodulin binding domain (CaMBD) (Fig. 2B). The mutation was confirmed by Sanger sequencing. No additional mutations in genes encoding for RBCs cytoskeletal, membrane or channel proteins were detected by whole exome sequencing in the affected patients.

### Functionality of the mutated Gardos channel

Gardos channel activity was evaluated measuring whole-cell currents by patch-clamp and K^+^(^86^Rb^+^) influx from mature RBCs.

**Figure 3:**
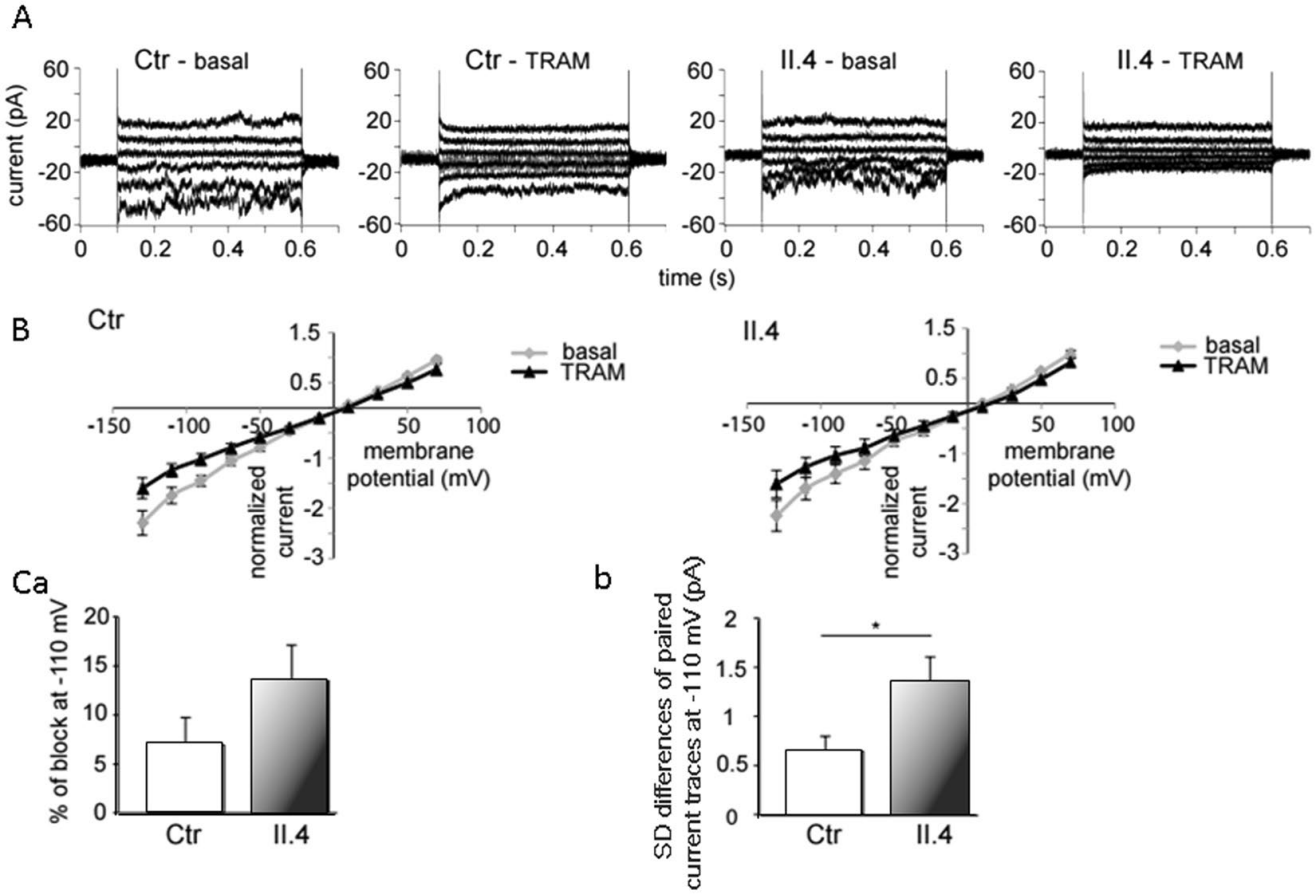
Whole-cell recordings of KCNN4 (Gardos channel) currents in RBCs from healthy donors and patient II.4. Currents were elicited by voltage steps from −130 mV to 70 mV for 500 ms in 20 mV increments at V_h_ = −30 mV and recorded in the absence and after application of 1 µM TRAM-34, a specific Gardos channel blocker. (**A**) Raw current traces from healthy and mutated RBCs in the absence (basal) and in the presence of 1 µM TRAM-34 as indicated above the recordings. For clarity, in all the panels, not all the traces, but every second one, starting with −130 mV, are being shown. (**B**) Corresponding I/V-curves in the absence of 1 µM TRAM-34, basal (grey diamonds) and in the presence of 1 µM TRAM-34 (black triangles) for healthy (Ctr) and mutated (II.4) RBCs. Data are expressed as mean current ± SEMs. (**C**) Differences in Gardos currents between healthy (n = 27 cells) and mutated RBCs (n = 23 cells) at −110 mV. (**Ca**) compares the percent of block (by 1 µM TRAM-34) of mean currents in healthy and mutated RBCs. Due to the small number and small single channel conductivity of Gardos channels in RBCs an additional assessment of Gardos currents is given by analysis of the kinetics of current traces. A mean whole-cell current being the result from the summation of many smaller unit currents flowing through single ion channels, exhibits fluctuations or “noise” about its mean level. (**Cb**) compares the difference in the standard deviation (SD) of current traces (with and without 1 µM TRAM-34) between control and patient RBCs. Significance was checked based on an unpaired t-test *p < 0.05.

Figure 3A–C summarizes the whole-cell measurements showing raw current traces (Fig. 3A), I-V-curves (Fig. 3B), a comparison of the percent block by 1 μM TRAM-34 of the current at −110 mV (control vs. patient II.4, Fig. 3Ca) and an assessment of the difference (with and without 1 μM TRAM-34) in the standard deviation (SD) of the current at −110 mV (control vs. patient II.4, Fig. 3Cb). Application of the Gardos channel blocker TRAM-34 reduces current amplitudes in RBCs of both healthy controls and patient II.4 (Fig. 3B). The effect of TRAM-34 is mostly pronounced at very negative voltages; this is fully compatible with the considerable inward rectification reported for the channel^14^ and was similarly detected in the comparison between RBCs of K_Ca_3.1-knock-out and wild type mice^15^.

As shown in Fig. 3Ca, the patient’s cells show a tendency for an increased percentage of current block by TRAM-34 as assessed by mean-currents, although not statistically significant (p = 0.08); the patient’s channel activity is clearly increased as judged by the comparison of the difference in the SD of current with and without TRAM-34, such a difference being a read-out for Gardos currents even in cells expressing a few channels (Fig. 3C). In the whole-cell configuration the cytosolic Ca^2+^ is equilibrated with the pipette solution, i.e. equal Ca^2+^-concentration for patient II.4 and healthy controls.

These data confirm that KCNN4 R352H mutation is a gain-of-function mutation leading to increased channel activity.

**Figure 4:**
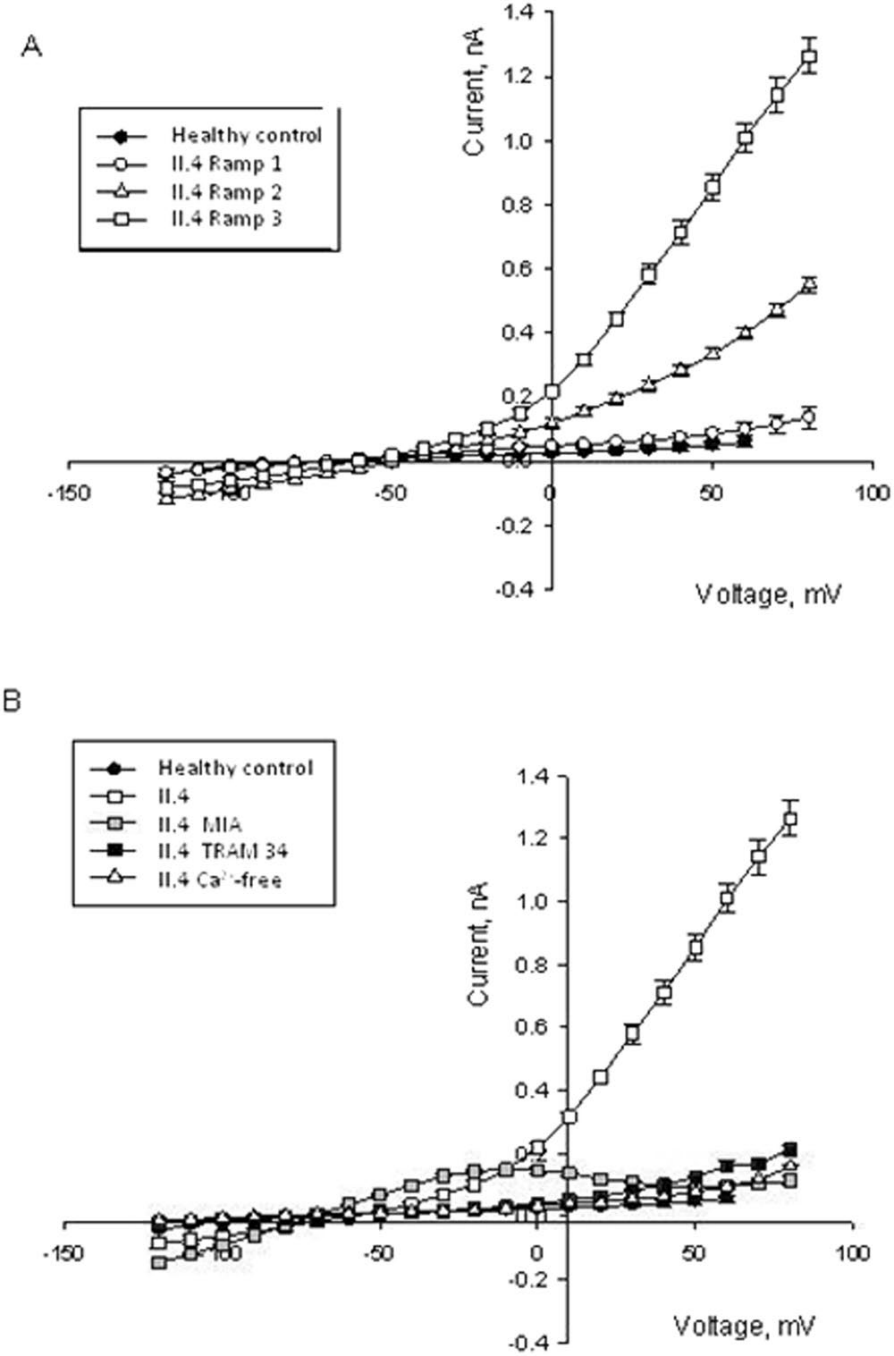
Current-voltage plots for the erythroid precursor cells of the healthy controls (n = 17, black circles) and II-4 patient (open or grey symbols). (**A**) Cells were exposed to a ramp protocol three times in a row. Each next hyperpolarization-depolarization cycle was followed by an increase in current at positive potentials in the EPCs of II.4 patient (n = 24, 19 and 12 for the ramps 1, 2 and 3), whereas for the cells derived from healthy donors it was never the case. (**B**) Increase in electric current at positive potential in cells of patient II.4 caused by repetitive ramp protocol application could be blocked by pre-treatment of cells with 15 µM methyl isobutyl amiloride (MIA, grey squares, n = 14), 15 µM TRAM-34 (black squares, n = 7) or omission of the extracellular Ca^2+^ (open triangles, n = 14). All I/V curves presented in the panel represent the values obtained for the third ramp protocol. Data are shown as mean ± SD.

Differences in current properties are not only characteristic of the circulating cells, but can also be tracked down to the polychromatic erythroblasts (EPCs) differentiated for 11–13 days *ex vivo* from the peripheral CD34+ cells. We detected an abnormal voltage-sensitivity of the Ca^2+^ and TRAM-34 sensitive current component in EPCs of the II.4 patient. Whereas erythroblasts produced from the CD34+ cells derived from healthy donors were insensitive to multiple depolarization-repolarization cycles, EPCs of patient II.4 responded to a repeated voltage steps protocol with a progressive increase in current within the positive potential range (Fig. 4). This increase in channel activity was associated with cell swelling that could be observed microscopically during the voltage-clamp recordings (data not shown). The depolarization-inducible current could be blocked by omission of Ca^2+^ from the extracellular solution, TRAM-34 supplementation, or addition of methyl isobutyl amiloride (MIA), an inhibitor of the Na^+^/H^+^-exchanger that prevented swelling in response to the ramp protocol application. These findings suggest an existence of a cross-talk between the Na^+^ uptake and swelling induced by the repeated changes in the transmembrane potential on one hand, and K^+^ loss through the Gardos channels on the other.

**Figure 5:**
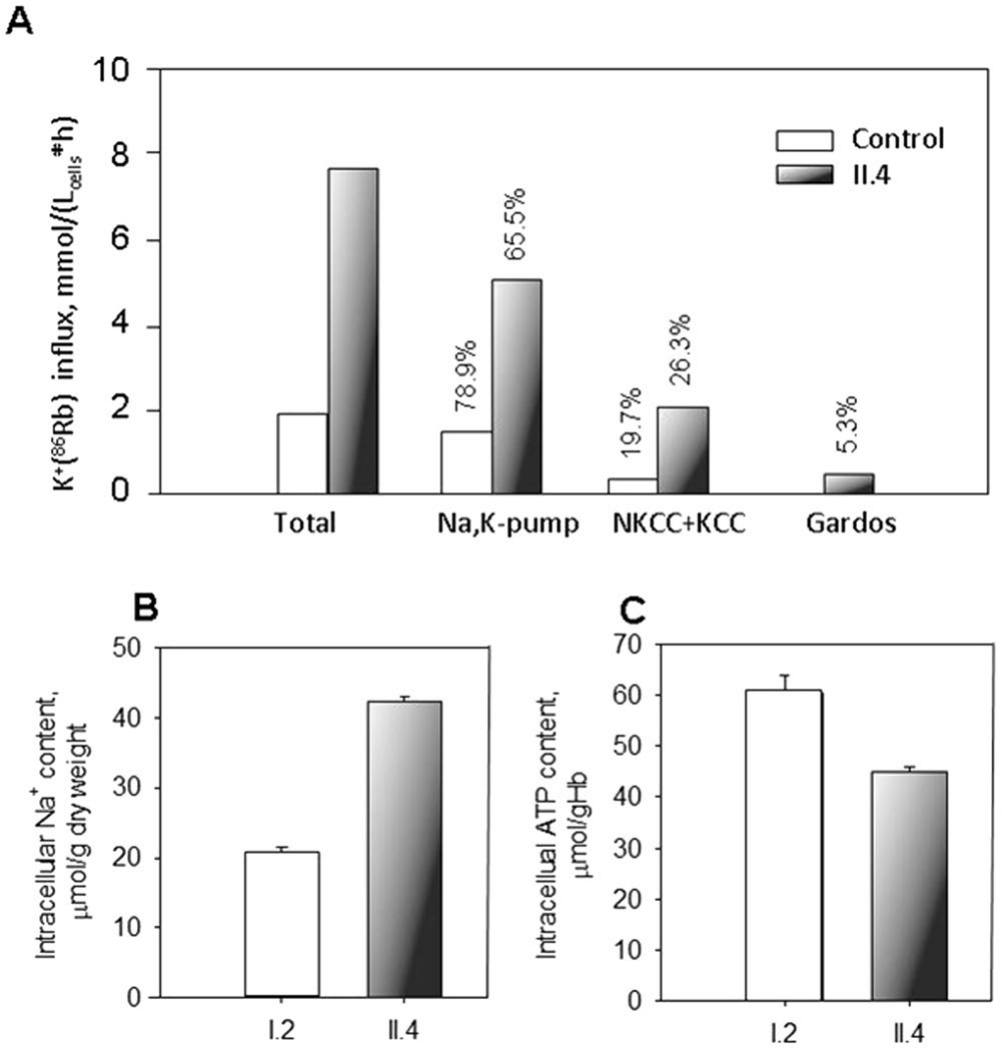
Activity of ion transporters, intracellular Na^+^ and ATP content in circulating RBCs. (**A**) Unidirecitonal K^+^(^86^Rb^+^) influx into RBCs of a healthy control and patient II.4. Shown in the panel are bulk K^+^ influx and the influx components carried by the Na,K-pump (ouabain-sensitive flux) and the fluxes mediated by the chloride-dependent influx mediated by Na,K,2Cl-, K,Cl-cotransporters, and Gardos channel activity (TRAM-34-sensitive flux). White bars show the flux values for RBCs of healthy control and black bars are for the fluxes in II.4 patient’s RBCs. Numbers above the bars indicate the contribution of each ion transporter into the total influx of either patient or healthy control in %. Intracellular Na^+^ (**B**) and ATP (**C**) content of RBCs of the healthy I.2 (white bar) and patient II.4 (black bar) measured in triplicates on one occasion. Data are mean ± SD. Although statistical analysis could not be performed for single experiments all the values show clear difference between the I.2 and II.4 (for intracellular Na^+^ and ATP levels) as well between the RBCs of II.4 and identically treated healthy control for all the K^+^(^86^Rb^+^) flux components.

We further extended our studies of ion movements through the RBC membrane to monitoring the unidirectional K^+^(^86^Rb^+^) influx in cells of II.4. The fraction of K^+^ uptake through the Gardos channel was detected as the flux sensitive to TRAM-34. This component was small, but detectable in RBCs of the II.4 patient, whereas it was below detection limits in the cells of the healthy donor (Fig. 5A).

### Activity of electroneutral transporters, Na,K-ATPase and glycolysis in mature RBCs

Hyper-activation of K^+^ flux through the Gardos channel in patient’s RBCs was accompanied by abnormally high activity of chloride-dependent K^+^ flux mediated by Na,K,2Cl- and K,Cl-cotransporters. Abnormally high passive K^+^ loss was compensated by a 4-fold increase in Na,K-ATPase activity in patient’s RBCs (Fig. 5A). Intracellular Na^+^ content in RBCs of II.4 was doubled when compared to the healthy control (Fig. 5B), despite the dramatic increase in the active Na^+^ extrusion by the Na,K-ATPase. Ion gradients in II.4 were maintained at a cost of ATP reduction (Fig. 5C), and stimulation of glycolysis in RBCs (could be observed as a facilitated depletion of plasma glucose and faster lactate production during 24 h of storage at room temperature (Glucose 2.45 vs. 3.20 mM and Lactate 8.25 vs. 5.6 mM in II.4 and control, respectively).

### Calcium handling in patients with mutated Gardos channel

**Figure 6:**
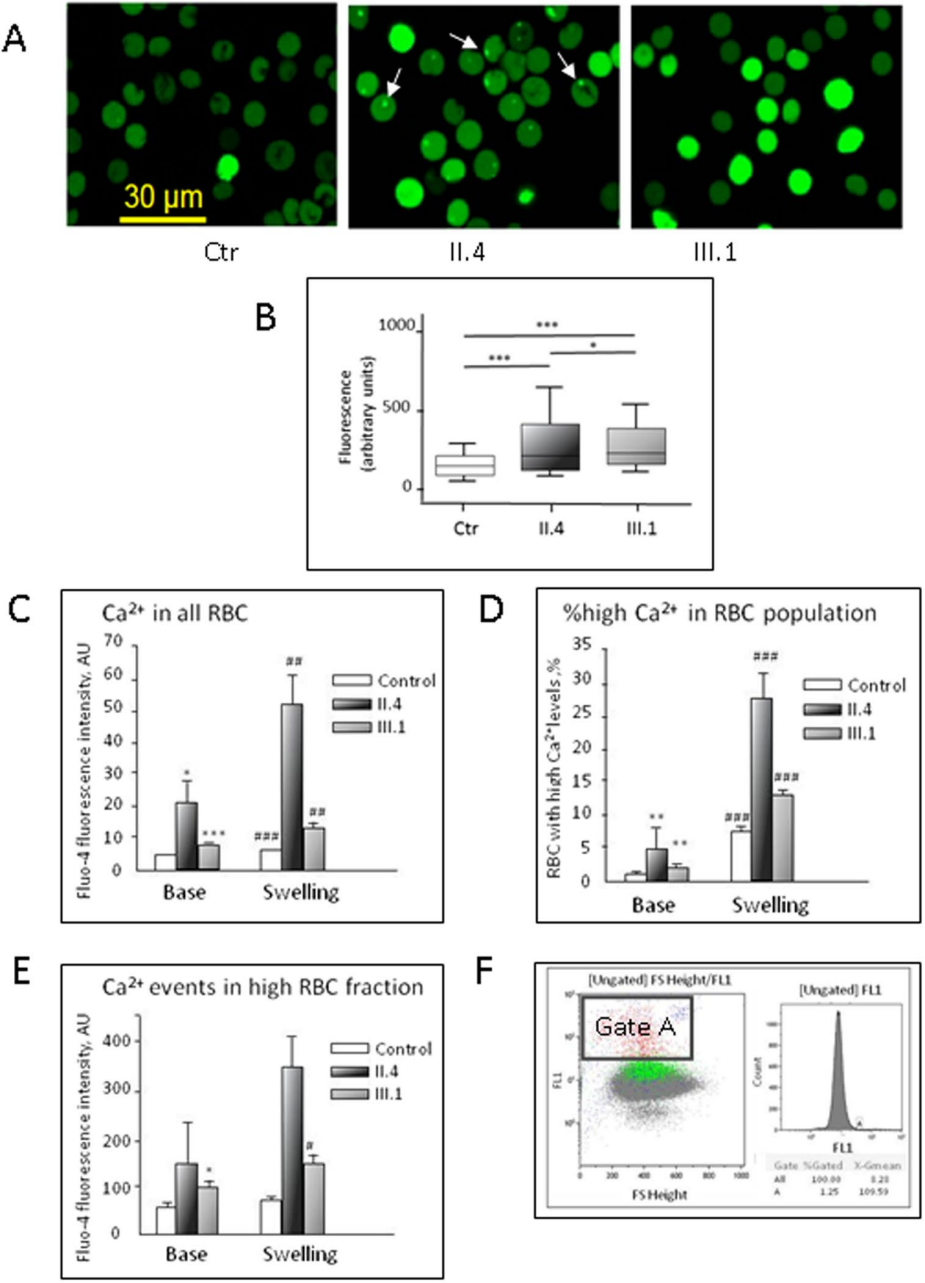
Calcium handling in patients with mutated Gardos channel. (**A**) Representative confocal images of RBCs from controls and the patients loaded with the Ca^2+^-sensitive dye Fluo-4. A brighter fluorescence corresponds to a higher Ca^2+^-concentration. White arrows indicate sequestration of Ca^2+^ in intracellular vescicles. (**B**) Statistical analysis of wide field fluorescence Fluo-4 recordings for healthy donors (white bar, n = 2495 cells), patient II.4 (black shaded bar, n = 1257 cells) and patient III.1 (grey bar, n = 1402 cells). Because values are not Gaussian distributed we plotted boxes with whiskers from the 10^th^ to 90^th^ percentile. Significance was checked using the Mann-Whitney test; *, ***denote p < 0.05, and 0.001 respectively. (**C**–**F**) Intracellular Ca^2+^ (assessed as Ca^2+^-dependent fluorescence of Fluo-4) in RBCs of healthy controls and patients II.4 and III.1 measured by flow cytometry. Intracellular Ca^2+^ was measured at baseline and then the measurements were repeated immediately after the initiation of osmotic swelling by adding a bolus of distilled water to dilute the RBC suspension in isosmotic buffer by 1/3 (Swelling). (**C**) Readout for all RBCs in suspension; (**D**) Amount of cells forming “high Ca^2+^ fraction” (A-gated fraction, flow cytometric analysis shown in panel (E); (**F**) fluorescence intensity of Fluo-4 in this “high Ca^2+^ fraction”. All the experiments were performed in two occasions, and each time triple measurements were performed for each conditions. *, ** and ***denote p < 0.05, 0.01 and 0.001 respectively compared to healthy control. ^#^, ^##^ and ^###^ stand for p < 0.05, 0.01 and 0.001 for osmotically compromised cells compared to the baseline values in either control or patients.

We measured intracellular Ca^2+^ levels in RBCs of II.4, III.1 and controls using fluorescence live imaging and flow cytometry. Figure 6A depicts the representative confocal images of fluo-4 fluorescence intensity proportional to the amount of free Ca^2+^. We observed an increased heterogeneity in the Ca^2+^ content and higher number of RBCs with extraordinary high free Ca^2+^ levels and eventual sequestration of Ca^2+^ in intracellular vesicles. Figure 6B shows statistical analysis of Ca^2+^ contents. The abnormally high and heterogeneous intracellular Ca^2+^ content was also confirmed by flow cytometry in both patients. The absence of splenic sequestration of more compromised cells in patient II.4 may justify the higher Ca^2+^ content respect to III.1. The observation of high intracellular Ca^2+^ hints to an increased activity of Ca^2+^-permeable ion channels such as PIEZO-1, the transient receptor potential cation channels (TRPC6), the voltage-dependent anion selective channels (VDACs), or N-methyl aspartate receptors (NMDARs), suggesting that me*c*hanical stimulation could be involved in tuning the activity of multiple ion transport pathways.

We therefore tested the sensitivity of Ca^2+^ uptake to mechanical stimulation (swelling). Hypoosmotic swelling was induced by acute supplementation of water to decrease the osmolarity to 220 mOsm. Intracellular Ca^2+^ was measured before and immediately after reduction of the osmolarity. As shown in Fig. 6(C–F), swelling triggered massive Ca^2+^ uptake in RBCs of both patients and controls. Intracellular free Ca^2+^ levels reached in the swollen cells of III.1 and, in particular, of II.4 substantially exceeded those measured in the RBCs of healthy subjects revealing abnormally high Ca^2+^ uptake in both patients.

These observations were in line with the sensitivity of the abnormally high ion currents in the EPCs of II.4 patient, not only to TRAM-34 and omission of Ca^2+^, but also to MIA that abolished swelling of EPCs in response to the repetitive ramp protocol application (Fig. 4B).

## Discussion

In this paper we report a new family with two members affected by chronic hemolytic anemia associated with the R352H mutation in the *KCNN4* gene encoding the Gardos channel.

Mutation R352H is located in the calmodulin binding domain of the Gardos channel. As also shown by Rapetti-Mauss *et al*.^10, 13^, the mutation not only changes the Ca^2+^ sensitivity affecting the channel threshold of activation, but modifies its functional properties *per se* resulting in a more active channel, the higher activity possibly due to altered open probability or unitary conductance.

The slightly bigger endogenous current observed in the case studied by Rapetti-Mauss *et al*.^13^, compared to our observations may be explained by differences in measurement protocols or sample handling, such as applying voltage ramps compared to discrete voltage steps, associated differences in the holding potential or RBC storage before performing the recordings^16^.

In this study we also show that differences in current properties are not only characteristic of the circulating RBCs, but can also be tracked down to erythroblasts differentiated *ex vivo* from peripheral patient CD34+ cells. The effects in precursor cells are more pronounced compared to mature circulating RBCs (Fig. 4 vs. Fig. 3), which is not surprising regarding the low abundance of the Gardos channel in mature circulating RBCs. Based on patch-clamp recordings Grygorczyk *et al*.^14^ estimated that approximately 75% of RBCs have between 1 and 5 channels per cell whereas the rest have approximately 11 to 55 channels per cell; Wolff *et al*.^17^ estimated an even lower number of an uniform mean of 1 to 3 channels per cell.

To get a mechanistic link between the Gardos channel R352H mutation, the cellular properties and eventually the clinical phenotype, we also measured the activity of single ion transporters using ^86^Rb^+^ as a tracer for K^+^ flux experiments, we evaluated the intracellular ions contents and the effects on RBC glycolysis. The observation of a 3–4 fold increase of unidirectional K^+^(^86^Rb^+^) fluxes in patient II.4 suggests that mutant R352H activate a series of compensatory processes resulting in the prevention of terminal dehydration.

**Figure 7:**
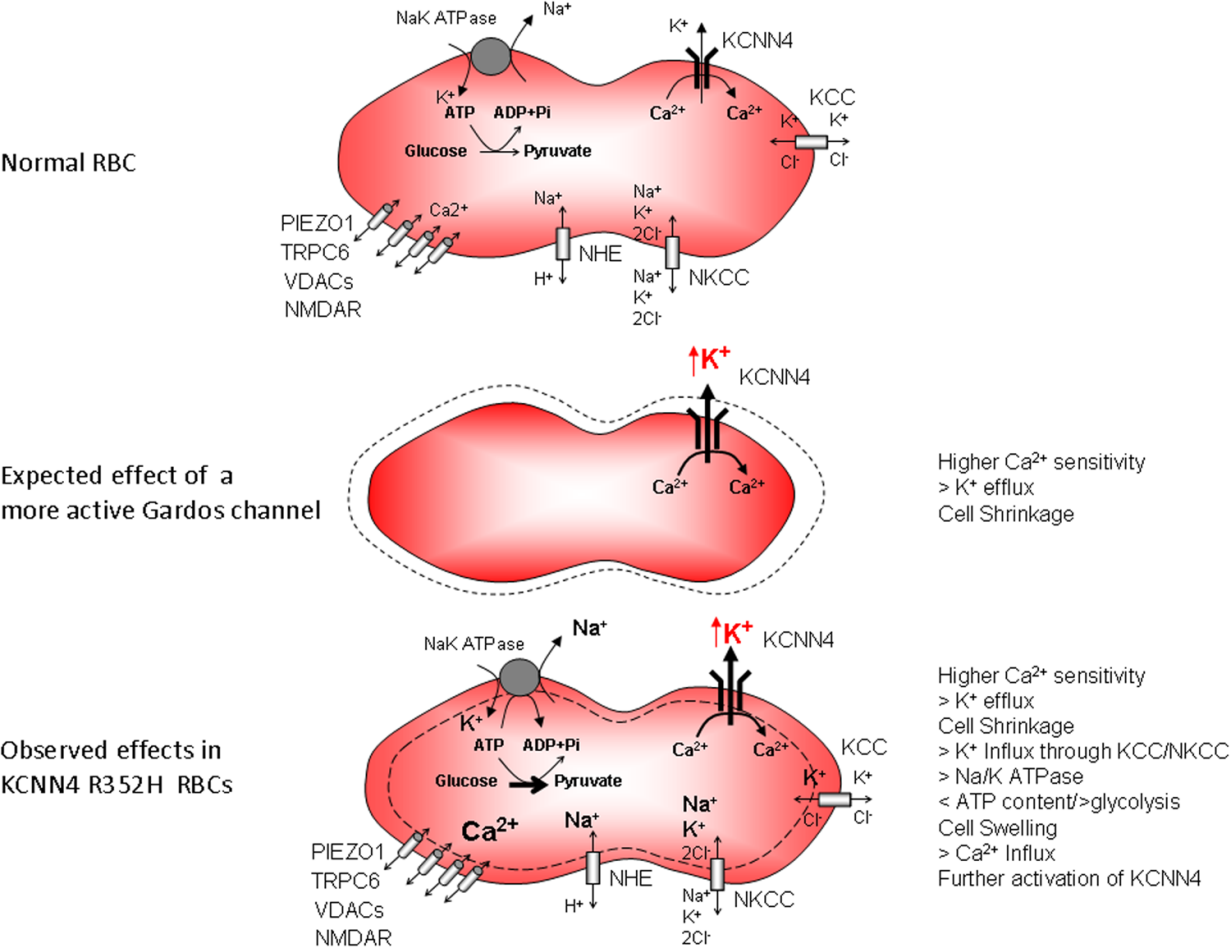
Schematic representation of the proposed mechanism observed in RBCs from patient carrying R352H *KCNN4* mutation. Top: Normal RBC; Middle: Expected effect of a more active Gardos Channel, Bottom: observed effects in KCNN4 R352H mutant RBCs.

A possible scenario is presented in the scheme reported in Fig. 7. Following the increased activity of the mutated Gardos channel and the subsequent excessive loss of cell K^+^, K^+^ influx through NKCC and possibly KCC, is enhanced. Moreover, the NKCC-mediated Na^+^ accumulation is further enhanced by activation of the Na^+^/H^+^ exchanger (NHE) as confirmed by our patch-clamp data on erythroid cells using NHE specific inhibitors and in line with the increase of the intracellular Na^+^ content observed in the splenectomized II.4 patient. The abnormally high intracellular Na^+^ content results in cell swelling, triggers activation of the Na,K-ATPase and increases energy consumption^18, 19^ as demonstrated by the ATP reduction and stimulation of glycolysis. Interestingly we found Ca^2+^ overload and increased heterogeneity of the inter- and intra-cellular distribution of Ca^2+^ in RBCs of both patients as assessed by flow cytometry and confocal fluorescence live-cell imaging. Such an overload could possibly be aided by activation of the mechanosensitive, thus swelling activated, PIEZO1 channels or through other Ca^2+^ entry pathways e.g. voltage-sensitive cation channels; these latter are possibly implied as observed by the increase of EPCs current as a result of repeated voltage ramps, then abolished by external Ca^2+^ removal.

Increase of the Ca^2+^ content in patients RBCs may trigger numerous Ca^2+^-dependent processes^20^ such as scramblase activation, calpain cleavage, flippase inhibition and ultimately provides a positive feedback loop for the Gardos channel itself. The markedly up-regulated intracellular Ca^2+^ levels in RBCs may contribute to premature removal of these RBCs from the circulation^21^. Thus based on our data, a point mutation in a single channel present in a low number of copies (3–200/cell according to different authors)^14, 17, 22^ in RBCs may interfere with the regulation of multiple ion transport systems and, consequently, with metabolic rates, membrane stability and cellular morphology of circulating RBCs as well as erythroblasts.

Recently, in a very short period of time, three different groups described mutations in the *KCNN4* gene associated with DHSt^10, 11, 12^; our report brings to 7 the number of affected families. The identification of R352H as a *de novo* event in patient II.4 reinforces the hypothesis that it could be a mutational hotspot^11^.

The patients with *KCNN4* mutations reported so far display a wide phenotypic heterogeneity, ranging from severe fetal anemia requiring *in utero* transfusions to mild or compensated hemolysis (Supplementary Table I). It is worth mentioning that all the 8 splenectomised patients do not display the increased susceptibility to thrombotic events commonly reported in DHSt^23, 24^; this holds especially true when considering the very long follow-up after splenectomy of patient II.4. An explanation for such an observation may reside in the increasingly recognized interplay between mechano-sensitive channels like PIEZO1 and the Gardos channel^3, 25^. When passing capillaries or other constrictions of smaller diameter, the RBCs mechano-sensitive channels that conduct Ca^2+^ are activated and the consecutive Ca^2+^-flashes activate Gardos channels for a fast and transient volume adaptation. This concept is supported by our measurements of filterability, since both inhibition of mechano-sensitive channels (with GsMTx-4) and inhibition of the Gardos channel (with TRAM-34) decrease filterability. Thus if the function of mechano-sensitive channels is impaired, such as Piezo1 in typical DHSt^26^, the above described volume adaptation does not work properly, RBCs can get stuck and form aggregates caused by intercellular adhesion^27^. In the presence of the spleen, RBCs lacking the adaptive volume regulation get stacked in the splenic slits and are consecutively removed, whereas in splenectomized patients they circulate leading to thrombotic events. In patients with the *KCNN4* mutation, Gardos channels are more sensitive to Ca^2+^ and more active, and with such compensation at hand, volume adaptation is less impaired suggesting a reduced post-splenectomy thrombotic risk, to be confirmed in further studies.

The dyserythropoietic features detected in the bone marrow of II.4 supports the hypothesis that KCNN4 may play a role in erythroid maturation, suggested also by the I-V-curves in precursor cells indicating a higher abundance of Gardos channels in erythroid precursors compared to mature RBCs; therefore, iron overload detected in our and other patients with *KCNN4* mutation^11^ may be a consequence of dyserythropoiesis, similar to what is reported in DHSt with *PIEZO1* mutations^28, 29^. The history of the patients reported here confirms that congenital hemolytic anemia due to abnormal cation permeability may represent a diagnostic challenge that is now overcome by means of the new Next Generation Sequencing technology approaches. Patient II.4 received a diagnosis of hereditary spherocytosis in infancy and was splenectomized without clinical improvement; the finding with SDS-PAGE of a spectrin deficiency may be considered a secondary effect of a membrane perturbation^30, 31^ disclosed by splenectomy^32^, since it is absent in the daughter, and no mutations in the spectrin or other related genes were detected by whole exome sequencing. The phenotypic variability, the possible high frequency of *de novo* variants, and the absence of specific biological tests make the diagnosis of this variant of hereditary stomatocytosis particularly difficult. The widespread use of NGS technology is likely to result in a greater than expected frequency of Gardos channelopathies.

In conclusion, this study shows that *KCNN4* R352H mutation determines, together with an increased Gardos channel activity, an unexpected activation of multiple compensatory changes, in absence of any other abnormalities of red cell membrane or channel proteins.

We suggest that the ‘Gardos channelopathy’ is a more complex pathology, to some extent different from the common DHSt type, and that distinct mutations in the Gardos channel may have a diverse impact on cell volume regulation mechanisms. In fact, as shown in Supplementary Table I, patients with R352H mutation are characterized by milder dehydratation and normal Osmoscan curve when compared to V282M/E cases that display typical features of hereditary xerocytosis^33, 34^, (median MCHC R352H 35 g/dL vs. MCHC V282M/E 36.4).

This observation is also in line with the recent study by Rapetti-Mauss *et al*. aimed to define the sensitivity of KCNN4 mutants to Senicapoc, where it was demonstrated that the three mutations (R352H, V282M/E) do not share a common mechanism in altering channel characteristics and display significantly different Senicapoc sensitivity^13, 35^. Further investigation of the molecular mechanisms behind the cross-talk between numerous ion transporters and exploration of the causes of the abnormal hyper-sensitivity to mechanic stimulation and of Ca^2+^ overload may give more clues to the optimization of diagnosis and therapeutic approaches for this group of patients.

## Methods

All the diagnostic procedures and investigations were performed in accordance with the Helsinki Declaration of 1975. Patients had given their consent for the participation to this study. The study was approved by Ethical Committee at Fondazione IRCCS Ca’ Granda Ospedale Maggiore of Milan.

### Hematologic investigations

Routine hematological investigations were carried out according to Dacie & Lewis^36^. RBC osmotic fragility was evaluated by NaCl test on fresh and incubated blood at 37 °C overnight, acidified glycerol lysis test (AGLT), and Pink test as described elsewhere^37^. EMA-binding was performed by the method of King *et al*.^38^, RBC enzymes’ activities were determined according to Beutler^39^, and membrane proteins were analyzed by SDS-PAGE^40, 41^.

Osmoscan was performed by Laser-assisted Optical Rotation Cell Analyzer (LoRRca MaxSis, Mechatronics, Hoorn, The Netherlands) following the manufacturer’s instructions; samples were tested in different experimental conditions (fresh blood, after 24 h at 4 °C) and drawn in Heparin and EDTA to ascertain whether incubation and anticoagulants may affect the curve as hypothesized by Badens & Guizouarn^42^. The Osmoscan curve performed after 24 h incubation at 37 °C was markedly altered in both patients and controls.

All the analyses were performed with cells of patient II.4, and of patient III.1 when feasible due to the limitation of blood sample size (child).

### Next Generation Sequencing

Library preparation, Whole Exome Sequencing and data analysis, performed before the report in literature of new mutations in KCNN4, were provided as service by Genomnia srl (Lainate, Italy), using the Life Technolgies SOLiD 5500xl Genetic Analysis Sequencer. For details please refer to Supplementary Information.

The mutation was confirmed by Sanger sequencing (ABI PRISM 310 Genetic Analyzer, Applied Biosystems).

### Isolation and culturing of erythroid precursor cells (EPCs)

Heparinised peripheral blood (PB) samples of patient II.4 and a healthy control were obtained and processed within 5 h after collection. Mononuclear cells were isolated on a Ficoll-Paque PLUS gradient (GE-Healthcare, Dietikon, Switzerland), and cultured in a two-phase liquid system as described elsewhere^43^. Late-stage erythroblasts were then used for assessment of electric currents across the EPCs membranes by means of patch-clamp.

### Filterability

The filterability test was performed with RBCs of patients II.4 and III.1 and controls by a modified method originally developed by Beutler *et al*.^44^ for the depletion of leukocytes. To assess the impact of mechano-sensitve channels and Gardos channel into filterability, healthy RBCs were treated with the blocker of mechano-sensitive channels (GsMTx-4) and the Gardos channel blocker TRAM-34 prior to the filtration onset. For a detailed description please refer to Supplementary Information.

### Separation of RBCs on Percoll density gradient

Separation of RBCs into the low (L), medium (M) and high (D) density fractions was performed as described elsewhere [21]. Whole blood (1–1.5 mL) was overlayed on 12.5 mL 90% isotonic Percoll solution (GE Healthcare; density 1.130 g/mL) prepared by mixing 90 mL of Percoll with 10 mL × 10 PBS (Sigma-Aldrich) and 11 mL × 1 PBS (Sigma-Aldrich). The resulting samples were centrifuged using Sorvall RC-5C plus centrifuge equipped with a SM-24 rotor at 4 °C for 30 min at 45,000× g.

### Patch-clamp measurements

Patch-clamp measurements on circulating RBCs were performed with a NPC-16 Patchliner (Nanion Technologies, Munich, Germany) and on the EPCs of patient II.4 with a conventional patch-clamp setup (Axon CNS, Molecular Devices, Downingtown, PA, USA) The resistance of the chips with internal and external solutions as follows (in mM): KCl 70, KF 70, HEPES 30, CaCl_2_ 0.5, pH = 7.2 adjusted with KOH (internal) and KCl 140, MgCl_2_ 5, CaCl_2_ 6, D-glucose 2.5, HEPES 10, pH = 7.3 adjusted with KOH (external) was between 5 and 8 MΩ. Gigaseals were considered successful if exceeding 5 GΩ (with most cells they were 10 GΩ and above). Gigaseal formation was facilitated by the use of a seal enhancing solution as recommended by the Patchliner manufacturer and containing (in mM): NaCl 80, KCl 3, MgCl_2_ 10, CaCl_2_ 35, HEPES 10, pH = 7.3 adjusted with NaOH. The seal enhancing solution was only used to help obtaining the very high gigaohmic contact between the cell and the chip and in the whole-cell configuration was replaced by the external solution. Whole-cell configuration was achieved by negative pressure suction pulses between −45 mbar and −150 mbar and its formation judged by the appearance of sharp capacitive transients. Whole-cell patch-clamp recordings were conducted at room temperature using voltage steps from −130 mV to 70 mV for 500 ms in 20 mV increments at 5 s intervals, the holding potential being set at −30 mV. Gardos current differences between patient II.4 and healthy donors were assessed by comparing the block of currents by 1 µM TRAM-34. To reduce inter-cell variability data are expressed as normalized current which is the ratio of the current under specified experimental conditions i.e. before (control) and in the presence of 1 µM TRAM-34 at selected membrane potentials, to the current at +70 mV determined 30–60 s before starting the control (control) measurement. Data are presented as means ± SEMs (n represents the number of cells).

Such an approach to avoid inter-cell variability we have successfully used^45^. A two-tailed, paired t-test was applied on the normalized currents to assess the difference in the current with and without 1 µM TRAM at every potential.

Assessment of the fluctuations of the current (obvious in Fig. 3A Ctrl-basal and II.4-basal and abolished by TRAM as seen in Ctrl-TRAM and II.4-TRAM) allowed us to account also for the cells with only a few channels. Analysis of the kinetics of the traces giving us a sensitive quantitative measure of the Gardos channel activity in all cells was done in the following way: we considered the variance of the current trace at −110 mV for each cell. From the variance we calculated the standard deviation for the trace (this was done separately for the trace without TRAM and for the trace with 1 μM TRAM). We then subtracted the two standard deviations (these are paired data of the same cell). This difference was considered as a read-out of the Gardos channel activity (more fluctuations = higher activity). 23 cells from the patient and 27 cells from the control were compared using a non-paired t-test (Fig. 3Cb).

Electric currents through the membranes of EPCs of a patient (II.4) were assessed as described elsewhere^46^. Whole-cell voltage clamp experiments were performed during *ex*-*vivo* hematopoiesis between day 11 and day 13. EPCs were plated on poly-L-lysine (0.01% vol/wt in H_2_O) coated cover glass slides and voltage-clamped at room temperature under constant perfusion with extracellular solution. For data acquisition and analysis the Axopatch 200B amplifier, Digidata 1440A, and pClamp 10.3 were used (all by Axon CNS, Molecular Devices, Downingtown, PA, USA).

The leak current was subtracted manually and the data were filtered at 5 kHz. A voltage-step protocol was used to asses the passive electrophysiological properties. The voltage was hold at −70 mV and stepped for 200 ms from −120 mV to +80 mV, in 10 mV increment. The protocol was repeated 1–3 times. The standard intracellular solution consisted of (in mM) 117 KCl, 11 HEPES, 1 EGTA, 10 NaCl, 2 MgCl_2_, 2 Na_2_ATP, adjusted to pH 7.2 with KOH was used. The standard extracellular solution consisted of (in mM) 135 NaCl, 5 KCl, 5 HEPES, 10 D-glucose, 1.5 CaCl_2_, adjusted to pH 7.35 with NaOH. When stated, extracellular solution was Ca^2+^-free and containing 1 mM EGTA or supplemented with the inhibitor of the Na^+^/H^+^ exchanger methyl isobuthyl amiloride (MIA) and blocker of Gardos channel TRAM-34 (final concentration for both blockers was 15 µM). To ensure fast drug application, a Rapid Solution Changer System was used, which allowed solution changes within 20 ms. Data are presented as means ± SEM.

### K^+^ (^86^Rb^+^) influx

Unidirectional influx rates for K^+^ were assessed by using ^86^Rb^+^ as a tracer as previously described^47^. Total unidirectional K^+^ influx was detected in erythrocytes incubated at hematocrit of 5–8% in the standard incubation medium containing (in mM): 145 NaCl, 4 KCl, 0.15 MgSO_4_, 2 CaCl_2_, 10 glucose, 10 sucrose, and 10 HEPES-Tris, 0.3 L-arginine, 0.3 L-glutamate, and 0.3 L-glycine, pH 7.4 at 37 °C. Chloride-free medium contained (in mM): 145 NaNO_3_ and 4 KNO_3_ instead of NaCl and KCl respectively. Ouabain (final concentration 100 µM), and TRAM-34 (10 µM) were used as selective blockers of the Na,K-ATPase, and the Gardos channel, respectively. Cells were pre-incubated for 15 min to enable binding of the inhibitors to their targets. Influx was then initiated and measured by addition of ^86^RbCl (10^4^ Bq/mL) and aliquots of the RBC suspension collected 10, 20, 30, 45, and 60 min after the onset of incubation. The cells were washed free from extracellular tracer, lysed and the amount of ^86^Rb^+^ accumulated in erythrocytes was measured and normalized to the amount of the radioactive tracer in the incubation medium. Ouabain-sensitive K^+^ influx was denoted as active K^+^ uptake by the Na,K-ATPase. Chloride-dependent K^+^ influx in the presence of ouabain represented cumulative activity of the Na-K-2Cl- and K-Cl-cotransporters. Treatment with TRAM-34 in the presence of ouabain resulted in the inhibition of K^+^ influx through the Gardos channels.

### Ca^2+^-imaging

The Ca^2+^ imaging was performed based on the Ca^2+^-fluorophor Fluo-4 (Life technologies, Carlsbad, CA, USA) using a set-up as previously described^48^. Basal Ca^2+^-related fluorescence was measured as described before^49^. To evaluate the cellular distribution, confocal images of Fluo-4 loaded RBCs were recorded on a TCS SP5 (Leica, Mannheim, Germany)^50^.

### Detection of the intracellular Ca^2+^ levels in RBCs by flow cytometry

Intracellular Ca^2+^ levels in RBC fractions of healthy control subjects as well as of II-4 and III-1 patients were detected by flow cytometry. 2 µL of whole blood suspended in the incubation medium was loaded Fluo-4 AM (Life technologies, Carlsbad, CA, USA). Thereafter, Ca^2+^-dependent fluorescence intensity was measured in RBCs using Gallios flow cytometer (Beckman Coulter). Triple measurements (100.000 cells/measurement) were performed and averaged for each condition.

Due to the RBCs marked heterogeneity in sensitivity to mechanical stimulation, and because Ca^2+^ uptake is acute and transient, swelling was chosen as the most appropriate approach to study RBCs sensitivity to mechanical stimulation.

Sensitivity of Ca^2+^ uptake to swelling was tested as follows. Basal intracellular Ca^2+^ levels in RBCs of patients and controls were measured and then the samples were diluted with distilled water (0.25 mL H_2_O added to 0.5 mL sample) and Ca^2+^ measurements repeated directly thereafter.

### Statistics

Unless otherwise stated statistical analysis was performed using one-way ANOVA with Bonferroni’s multiple comparison test or Student’s paired *t*-test, as appropriate (with normal distribution of the values), Kruskal-Wallis with Dunn’s post test (when the distribution was not normal). For all tests, significance level was set at *P* < 0.05.

## Electronic supplementary material

supplementary materials

## References

[1] Maher AD, Kuchel PW (2003). The Gárdos channel: a review of the Ca2+-activated K+ channel in human erythrocytes. Int J Biochem Cell Biol.

[2] Fanger CM (1999). Calmodulin mediates calcium-dependent activation of the intermediate conductance KCa channel, IKCa1. J Biol Chem.

[3] Dyrda A (2010). Local membrane deformations activate Ca2+-dependent K+ and anionic currents in intact human red blood cells. PLoS One.

[4] Wulff H, Köhler R (2013). Endothelial small-conductance and intermediate-conductance KCa channels: an update on their pharmacology and usefulness as cardiovascular targets. J Cardiovasc Pharmacol..

[5] Wulff H, Castle NA (2010). Therapeutic potential of KCa3.1 blockers: recent advances and promising trends. Expert Rev Clin Pharmacol.

[6] Ataga KI (2011). Improvements in haemolysis and indicators of erythrocyte survival do not correlate with acute vaso-occlusive crises in patients with sickle cell disease: a phase III randomized, placebo-controlled, double-blind study of the Gardos canne blocker senicapoc (ICA-17043). Br J Haematol.

[7] Ataga KI, Stocker J (2009). Senicapoc (ICA-17043): a potential therapy for the prevention and treatment of hemolysis-associated complications in sickle cell anemia. Expert Opin Investig Drugs.

[8] Ataga KI (2008). Efficacy and safety of the Gardos channel blocker, senicapoc (ICA-17043), in patients with sickle cell anemia. Blood.

[9] Hoffman JF (2003). The hSK4 (KCNN4) isoform is the Ca2+-activated K+ channel (Gardos channel) in human red blood cells. Proc Natl Acad Sci USA.

[10] Andolfo I (2015). A. Novel Gardos channel mutations linked to dehydrated hereditary stomatocytosis (Xerocytosis). Am J Hematol.

[11] Glogowska E (2015). Mutations in the Gardos channel (KCNN4) are associated with hereditary xerocytosis. Blood.

[12] Rapetti-Mauss R (2016). Senicapoc: a potent candidate for the treatment of a subset of Hereditary Xerocytosis caused by mutations in the Gardos channel. Haematologica.

[13] Grygorczyk R (1984). Ca2+-activated K+ channels in human red cells. Comparison of single-channel currents with ion fluxes. Biophys J.

[14] Föller M (2010). Functional significance of the intermediate conductance Ca2+-activated K+ channel for the short-term survival of injured erythrocytes. Pflugers Arch.

[15] Rapetti-Mauss R (2015). A mutation in the Gardos channel is associated with hereditary xerocytosis. Blood.

[16] Makhro A (2016). Red Cell Properties after Different Modes of Blood Transportation. Front Physiol.

[17] Wolff D, Cecchi X, Spalvins A, Canessa M (1988). Charybdotoxin blocks with high affinity the Ca-activated K+ channel of Hb A and Hb S red cells: individual differences in the number of channels. J Membr Biol.

[18] Blanco G, Mercer RW (1998). Isozymes of the Na-K-ATPase: heterogeneity in structure, diversity in function. Am J Physiol.

[19] Hoffman JF (2002). Na pump isoforms in human erythroid progenitor cells and mature erythrocytes. Proc Natl Acad Sci USA.

[20] Bogdanova A (2013). Calcium in red blood cells-a perilous balance. Int J Mol Sci.

[21] Lutz HU, Bogdanova A (2013). Mechanisms tagging senescent red blood cells for clearance in healthy humans. Front Physiol.

[22] Lew VL (1982). Properties of the Ca2+-activated K+ channel in one-step inside-out vesicles from human red cell membranes. Nature.

[23] Stewart GW (1996). Thrombo-embolic disease after splenectomy for hereditary stomatocytosis. Br J Haematol.

[24] Delaunay J (2004). The hereditary stomatocytoses: genetic disorders of the red cell membrane permeability to monovalent cations. Semin Hematol.

[25] Cahalan SM (2015). Piezo1 links mechanical forces to red blood cell volume. Elife.

[26] Gallagher PG (2013). Disorders of red cell volume regulation. Curr Opin Hematol.

[27] Kaestner L (2012). Lysophosphatidic acid induced red blood cell aggregation *in vitro*. Bioelectrochemistry.

[28] Imashuku S (2016). PIEZO1 gene mutation in a Japanese family with hereditary high phosphatidylcholine hemolytic anemia and hemochromatosis-induced diabetes mellitus. Int J Hematol.

[29] Del Orbe Barreto R (2016). Hereditary xerocytosis, a misleading anemia. Ann Hematol.

[30] Fortier N (1988). The relationship between *in vivo* generated hemoglobin skeletal protein complex and increased red cell membrane rigidity. Blood.

[31] Salomao M, Chen K, Villalobos J, Mohandas N, An X, Chasis JA (2010). Hereditary spherocytosis and hereditary elliptocytosis: aberrant protein sorting during erythroblast enucleation. Blood.

[32] Mariani M (2008). Clinical and hematologic features of 300 patients affected by hereditary spherocytosis grouped according to the type of the membrane protein defect. Haematologica.

[33] Glader BE, Fortier N, Albala MM, Nathan DG (1974). Congenital hemolytic anemia associated with dehydrated erythrocytes and increased potassium loss. N Engl J Med.

[34] Grootenboer S (2000). Pleiotropic syndrome of dehydrated hereditary stomatocytosis, pseudohyperkalemia, and perinatal edema maps to 16q23-q24. Blood.

[35] Jensen BS, Hertz M, Christophersen P, Madsen LS (2002). The Ca2+-activated K+channel of intermediate conductance: a possible target for immune suppression. Expert Opin Ther Targets.

[36] Dacie, J. V. & Lewis, S. M. Practical Haematology. 9th edn., Churchill Livingston, London (2001).

[37] Bianchi P (2012). Diagnostic power of laboratory tests for hereditary spherocytosis: a comparison study in 150 patients grouped according to molecular and clinical characteristics. Haematologica.

[38] King MJ (2000). Rapid flow cytometric test for the diagnosis of membrane cytoskeleton-associated haemolytic anaemia. Br J Haematol.

[39] Beutler, E. Red cell metabolism: a manual of biochemical methods. New York, NY, Grune & Stratton, Inc. (1984).

[40] Fairbanks G, Steck TL, Wallach DF (1971). Electrophoretic analysis of the major polypeptides of the human erythrocyte membrane. Biochemistry.

[41] Laemmli UK (1970). Cleavage of structural proteins during the assembly of the head of bacteriophage T4. Nature.

[42] Badens C, Guizouarn H (2016). Advances in understanding the pathogenesis of the red cell volume disorders. Br J Haematol.

[43] Makhro A (2013). N-methyl-D-aspartate receptors in human erythroid precursor cells and in circulating red blood cells contribute to the intracellular calcium regulation. Am J Physiol Cell Physiol.

[44] Beutler E, West C, Blume KG (1976). Removal of Leukocytes and Platelets From Whole-Blood. J Lab Clin Med.

[45] Petkova-Kirova P (2000). 4-aminopyridine affects rat arterial smooth muscle BK(Ca) currents by changing intracellular pH. Br J Pharmacol..

[46] Hänggi P (2015). Functional plasticity of the N-methyl-d-aspartate receptor in differentiating human erythroid precursor cells. Am J Physiol Cell Physiol.

[47] Makhro A (2010). Functional NMDA receptors in rat erythrocytes. Am J Physiol Cell Physiol.

[48] Wang J (2013). Morphologically homogeneous red blood cells present a heterogeneous response to hormonal stimulation. PLoS One.

[49] Wang J, van Bentum K, Sester U, Kaestner L (2014). Calcium homeostasis in red blood cells of dialysis patients in dependence of erythropoietin treatment. Front Physiol.

[50] Flormann D (2015). Is there a role of C-reactive protein in red blood cell aggregation?. Int J Lab Hematol.

