# Supplementary material for: ‘Gardos Channelopathy’: a variant of hereditary Stomatocytosis with complex molecular regulation
Source: Sci Rep. 2017 May 11;7:1744. doi: 10.1038/s41598-017-01591-w (PMC5431847; doi:10.1038/s41598-017-01591-w)
Supplement: Supplementary file 1 — supplementary materials [file 41598_2017_1591_MOESM1_ESM.doc]

**Gardos Channelopathy’: a variant of hereditary Stomatocytosis with complex molecular regulation**

Elisa Fermo, PhD^1^, Anna Bogdanova, PhD^2^, Polina Petkova-Kirova PhD^3^, Anna Zaninoni PhD^1^, Anna Paola Marcello PhD^1^, Asya Makhro PhD^2^, Pascal Hänggi PhD^2^, Laura Hertz MSc^3^, Jens Danielczok ^3^, Cristina Vercellati PhD^1^, Nadia Mirra MD^4^, Alberto Zanella MD^1^, Agostino Cortelezzi, MD^1,5^, Wilma Barcellini, MD^1^, Lars Kaestner, PhD^6,7^, Paola Bianchi, PhD^1*^.

1 UOC Oncoematologia, UOS. Fisiopatologia delle Anemie Fondazione IRCCS Ca’ Granda Ospedale Maggiore Policlinico, Milano, Italy

2 Vetsuisse Faculty and the Zurich Center for Integrative Human Physiology (ZIHP), Institute of Veterinary Physiology, University of Zurich, Zurich, Switzerland

3 Research Center for Molecular Imaging and Screening, Medical School, Institute for Molecular Cell Biology, Saarland University, Homburg/Saar, Germany

4 UOC Pronto soccorso, Pediatria ambulatoriale e DH/MAC. Fondazione IRCCS Ca’ Granda Ospedale Maggiore Policlinico, Milano, Italy

5 Universita’ degli Studi di Milano, Italy

6 Experimental Physics, Saarland University, Saarbruecken, Germany

7 Theoretical Medicine and Biosciences, Saarland University, Homburg/Saar, Germany

**SUPPLEMENTARY METHODS**

*Next Generation Sequencing*

Sequencing libraries were prepared using SureSelect XT Target Enrichment system for SOLiD 5500 Multiplexed Sequencing, version A0, October 2012 (Agilent Technologies, Santa Clara, CA, USA). Exon capture was performed using the SureSelect XT Human All Exon V5 kit (Agilent). The quality of the enriched libraries was evaluated using the 2100 Bioanalyzer (Agilent). An equimolar pool of the barcoded libraries was prepared and amplified onto beads using emulsion PCR following SOLiD® EZ Bead™ system protocols (Life Technologies). Templated beads were deposited on slides, around 60M/library, and forward sequenced at 75bp reads length with Exact Call Chemistry Module (ECC) using the Life Technolgies SOLiD 5500xl Genetic Analysis Sequencer.

The sequencing reads were mapped and analyzed with the Lifetech Lifescope suite (release 2.5.1), using GRCh38 as a target genome and the annotation file for the enrichment kit Agilent SureSelect AllExon V5. SNVs or Indels were characterized with the Ensembl Variant Effect Predictor tool, using Sift and Polyphen to evaluate the predicted effect on protein functionality where feasible. The candidate variant in the *KCNN4* gene was identified by selecting variants shared by the two probands and absent in the healthy relatives. The PFAM website was used to identify the affected protein domain. The expression profile of the orthologue gene in murine primitive and definitive erythroid cells at progressive stages of maturation was extracted from ErytrhonDB.

*Filterabilty*

Blood samples were centrifuged at 1000xg for 20 min. Plasma was aspirated and mixed with phosphate buffered saline (PBS) (1:10). Erythrocytes were mixed with Tyrode solution (1:1) and washed 3 times (1000xg, 5 min). Filter paper (Whatman No. 4 GE Healthcare, UK) was pressed in a 3 mL syringe (Omnifix Solo Lure, Braun, Germany) and 200 mg Sigma- und 100 mg Alpha-Cellulose was added. The syringe was filled further with 2 mL Tyrode solution and shaken to allow the cellulose to mix. After the Tyrode drained, the syringe was primed with 2 mL of the diluted plasma. 500 µL of the RBC/Tyrode mixture was added at stopped flow conditions. Another 2 mL of Tyrode solution was carefully added. The flow through the syringe was started and the filtrate collected for exactly one minute. The amount of RBCs was related to the amount of hemoglobin, which was determined photometrically. To standardize the measurements different forms of hemoglobin were converted to hemiglobincyanid as previously described^1^. Absorption was measured at 546 nm (Lambda Bio+, Perkin Elmer, Waltham, MA, USA).

**Supplementary Figure 1**: Light microscopy of peripheral blood (PB) and bone marrow (BM), 100X. A: II.4 PB; B: III.1 PB; C, D: II.4 BM. Both patients displayed marked anisopoikilocytosis and stomatocytes. Detailed % of abnormal RBCs is reported in Table I. Bone marrow examination in II.4 revealed erythroid hyperplasia with some dyserythropoietc changes, in particular binucleated erythroblasts.


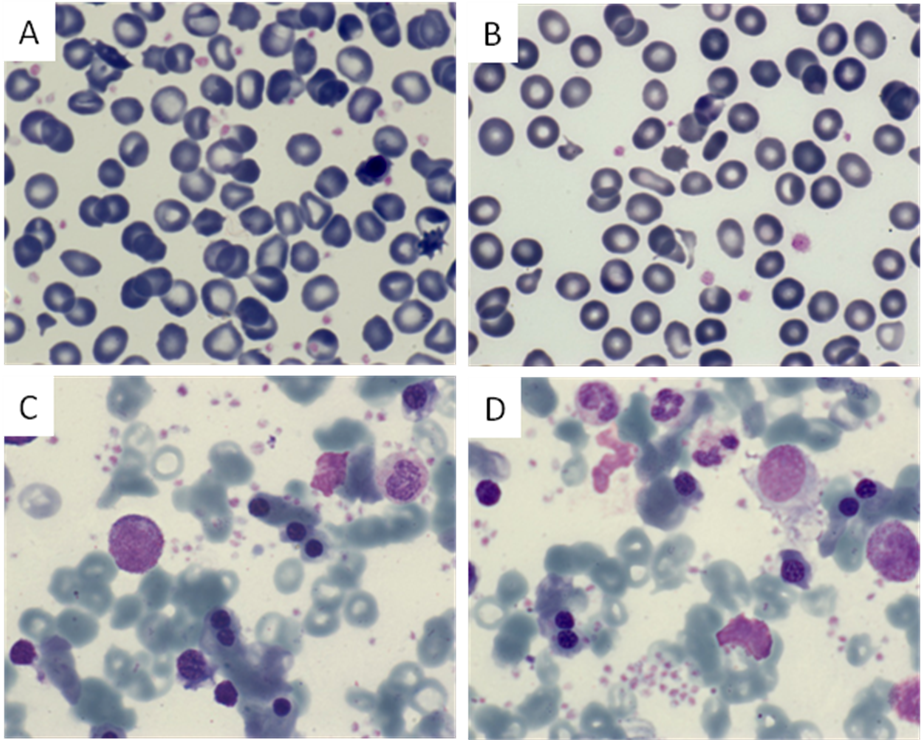


**References:**

1. Meyer-Wilmes, R. & Remmer, H. Die Standardisierung des roten Blutfarbstoffes durch Haemiglobincyanid. Naunyn - *Schmiedebergs Arch* **229** (1956).

**Supplementary Table I.** Clinical and hematologic data of patients so far reported in literature grouped according with the kind of KCNN4 mutations

| **Mutation** | **Patient** | **Anemia** | **Transfusion requirment#** | **Splenect. (years)** | **Stomato-cytes** | **Hb (g/dL)** | **MCV**  **(fL)** | **MCHC**  **(g/dL)** | **Retics Abs no** | **Ferritin** | **Osmotic fragility** | **Ema- binding** | **Ektacytometer** |
| --- | --- | --- | --- | --- | --- | --- | --- | --- | --- | --- | --- | --- | --- |
| **R352H** |  |  |  |  |  |  | 80-100 | 31-35 |  |  |  |  |  |
| Rapetti Mauss 2015 | 1-IV-3* | in utero anemia & Tx | 2 (infancy) | No | rare | 9.8 | 87.9 | 35.6 | 263 | 116 | << at 37° | Normal | Normal |
|  | 1-III-3 | moderate in childhood | 1 (infancy) | yes (25) | rare | 8.5 | 109 | 35.4 | 255 | 94 | << at 37° | Normal | Normal |
|  | 2-II-1** | moderate in childhood | 3 | No | rare | 11 | 93.1 | 36.1 | 249 | nd |  | Normal | Normal |
|  | 2-III-1 | mild since birth | 3 | No | rare | 10.4 | 86.9 | 36.5 | 363 | 121 |  | Normal | Normal |
| Andolfo 2015 | NA-II.3 | since age of 7 years | 3 | yes (21) | several | 10.4-9 | 101-112 | 34-31.6 | 358-450 | 580 | Normal | Normal | Slight R-shift |
|  | NA-III.1 | since birth | 3 | No | rare | 10.5 | 86 | 33.5 | 229 | 132 | Normal | Normal | Slight L-shift |
| This study | II.4 | since birth | 2 (u.s.) | yes (11) | 11% | 10.5 | 108 | 33 | 384 | 1108 | Normal | Normal | Normal |
|  | III.1 | since birth | 2 (infancy) | No | 8% | 9.9 | 81.5 | 35.7 | 273 | 167 | Slightly > | Normal | Normal |
| **V282M** |  |  |  |  |  |  |  |  |  |  |  |  |  |
| Andolfo 2015 | WO-IV.5 | mild | n.a. | n.a. | n.a. | 12.5 | 96 | 36.1 | 403 | n.a. | n.a. | n.a. | L-shift |
|  | WO-III.5 | mild | n.a. | n.a. | n.a. | 13.3 | 103 | Nd | 254 | n.a. | n.a. | n.a. | L-shift |
| Glogowska 2015 | kindred1 | n.a |  |  |  |  |  |  |  |  |  |  |  |
| **V282E** |  |  |  |  |  |  |  |  |  |  |  |  |  |
| Glogowska 2015 | kindred2 | since infancy | 1 (u.s) | yes (6) | yes | 10 | 106 | 36.4 | 25% | n.a. | << | n.a. | n.a. |
|  | (son) | since infancy | n.a. | No | yes | 9.8 | 103 | 36.8 | 27% | n.a. | << | n.a. | n.a. |
|  |  |  |  |  |  |  |  |  |  |  |  |  |  |
| *Other 4 family members reported with similar clinical pictures, three of them splenectomized. | | | | | | | |  |  |  |  |  |  |
| ** the father referred with anemia and splenectomized | | | |  |  |  |  |  |  |  |  |  |  |
| # Transfusion requirement: 1- regular until splenectomy or adolescence; 2- occasional; 3 - never transfused; u.s. –until splenectomy | | | | | | | | | | | |  |  |
| n.a. not available | | |  |  |  |  |  |  |  |  |  |  |  |
|  | | |  |  |  |  |  |  |  |  |  |  |  |
|  | | |  |  |  |  |  |  |  |  |  |  |  |
|  |  |  |  |  |  |  |  |  |  |  |  |  |  |
